# Clonal Integration Enhances the Performance of a Clonal Plant Species under Soil Alkalinity Stress

**DOI:** 10.1371/journal.pone.0119942

**Published:** 2015-03-19

**Authors:** Wenjun Zhang, Gaowen Yang, Juanjuan Sun, Jishan Chen, Yingjun Zhang

**Affiliations:** 1 Department of Grassland Science, China Agricultural University, Beijing, China; 2 College of Agro-grassland Science, Nanjing Agricultural University, Nanjing, China; Brigham Young University, UNITED STATES

## Abstract

Clonal plants have been shown to successfully survive in stressful environments, including salinity stress, drought and depleted nutrients through clonal integration between original and subsequent ramets. However, relatively little is known about whether clonal integration can enhance the performance of clonal plants under alkalinity stress. We investigated the effect of clonal integration on the performance of a typical rhizomatous clonal plant, *Leymus chinensis*, using a factorial experimental design with four levels of alkalinity and two levels of rhizome connection treatments, connected (allowing integration) and severed (preventing integration). Clonal integration was estimated by comparing physiological and biomass features between the rhizome-connected and rhizome-severed treatments. We found that rhizome-connected treatment increased the biomass, height and leaf water potential of subsequent ramets at highly alkalinity treatments but did not affect them at low alkalinity treatments. However, rhizome-connected treatment decreased the root biomass of subsequent ramets and did not influence the photosynthetic rates of subsequent ramets. The biomass of original ramets was reduced by rhizome-connected treatment at the highest alkalinity level. These results suggest that clonal integration can increase the performance of clonal plants under alkalinity stress. Rhizome-connected plants showed dramatically increased survival of buds with negative effects on root weight, indicating that clonal integration influenced the resource allocation pattern of clonal plants. A cost-benefit analysis based on biomass measures showed that original and subsequent ramets significantly benefited from clonal integration in highly alkalinity stress, indicating that clonal integration is an important adaptive strategy by which clonal plants could survive in local alkalinity soil.

## Introduction

Clonal plants are widely distributed in terrestrial ecosystems [1] and can survive in stressful habitats, because they can transport resources such as carbohydrates, water and nutrients from original ramets growing in non-stressful habitats to subsequent ramets in stressful conditions through a connecting rhizome or stolons [2–3]. The biomass of subsequent ramets with rhizome connections might be higher than plants where rhizomes were severed because of clonal integration [4–5]. Numerous studies showed that clonal integration could support subsequent ramets to survive in stressful environments, e.g. highly salinity [6–7], drought [8] and nutrient-depleted habitats [2, 9]. Original ramets located in lower salinity can transporte water and carbon products to subsequent ramets in higher salinity soil to enhance the performance of the connected subsequent ramets [10]. However, little is known about how clonal integration influences the performance of clonal plants under soil alkalinity stress. Soil alkalization has become a widespread environmental problem and is an important limiting factor for plant productivity [11–12]. Alkalinity influences plant morphology, growth and reproduction, and high pH can directly affect root growth [13].

Phenotypic plasticity is the ability of a genotype to modify its growth and development in response to environmental changes [14]. Clonal plants in heterogeneous environments can enable ramets to enhance the capture of locally abundant resource by allocating more photosynthate resources to roots in the high nutrient environment and more photosynthate resources to shoots in environments with high light [15–16]. In stressful environments, clonal plants allocate more resource to those organs that are currently impacted by the damage, such as more resources to leaves in herbivore-rich environments [17]. Clonal integration can buffer clonal plants against heterogeneous habitat by sharing resources through the structures such as rhizomes or stolons [18]. Nonetheless, how clonal integration influences clonal organs such as roots, rhizomes and buds under various levels of alkalinity stress remains unclear. Root is one of the most important component, and also is the main alimentative structure of growth and metabolism for plant [19], while rhizome and buds were believed to be very important clonal organs for plant reproduction. Based on plastic defense regulation [17], we expected that ramets might change the growth of root, rhizome or bud in stressful environments. For instance, clonal integration may enhance rhizome length and number of rhizome buds to facilitate escape from sites where stress level is intense and decrease root biomass allocation.

Clonal integration between ramets is normally found to occur in heterogeneous environments [20]. Previous studies showed that connected ramets had a greater clonal integration tendency in heterogeneous compared to homogeneous environments [21–22]. Maintaining clonal integration between ramets could have little significance for plants [23] as clonal integration may lead to the transportation costs [24]. Some studies showed that clonal integration could greatly improve survival and growth of subsequent ramets subjected to environmental stress, and the growth of original ramets in favorable conditions was not significantly reduced [16, 25]. However, other studies showed the performance of original ramets was reduced following clonal integration [26–27]. Original ramets in non-stressful and abundant habitats can serve as “donors” supplying resources, while subsequent ramets in stressful habitats can function as “recipients” obtaining resources [18]. Thus, the resource demand of subsequent ramets is probably related to their stress level. To reveal the cost and benefit of clonal integration, several studies have focused on the cost-benefit analysis, with measurements of plant biomass after connecting or severing rhizomes between original and subsequent ramets [17, 28]. The ecological viability of resistance as an efficient defense strategy depends on the balance of cost and benefit associated with plastic defense mechanisms [17]. Analysis of the cost-benefit of a given defense is a prerequisite for estimating its advantages and disadvantages and in turn for understanding the potential selection pressures that lead to the evolution of plant defenses [29].

*Leymus chinensis* is a rhizome clonal grass which is widely distributed in the Eurasian steppe [30] and was selected as the model species in our study. We evaluated the clonal integration through comparing physiological and biomass features between rhizome-connected and rhizome-severed treatments under four levels of soil alkalinity. Our aim was to address three questions: (1) How does clonal integration influence the performance of clonal plants under various levels of alkalinity stress? (2) How does clonal integration influence clonal organs such as roots, rhizomes and buds under various levels of alkalinity stress? (3) How does soil alkalinity affect the cost and benefit of clonal integration?

## Materials and Methods

### Plant materials

*L*. *chinensis* is a perennial species of Gramineae distributed in the eastern region of the Eurasian steppe zone, including the outer Baikal area of Russia, the northern and eastern parts of the People’s Republic of Mongolia, the Inner Mongolia Plateau, and the Songnen plain in northeast China. The species can adapt to medium salinity-alkalinity, drought, and low fertility stress [31]. *L*. *chinensis* with high palatability for livestock is often used as hay [32–33]. *L*. *chinensis* has long, strong rhizomes and its vegetative propagation is vigorous, giving rise to extensively spreading clones that often form mono-dominant plant communities in the steppe. Rhizomes of *L*. *chinensis* lie horizontally about 10 cm below the surface and are highly branched, with reproduction achieved mainly by clonal propagation [33].

Seeds of *L*. *chinensis* originated from their native grassland were germinated and pre-grown in soil collected from the native grassland (46°32′17″N, 125°28′24″E). The selected soil was collected from meadows having alkaline soil (pH = 8.9) with the permission from the Heilongjiang Grass Industry Research Institute of Agricultural Sciences in China. *L*. *chinensis* is not an endangered or protected species. *L*. *chinensis* was cultivated during March 2013 in a greenhouse in China Agricultural University, Beijing, China (39°54′27″N, 116°23′17″E) with air temperature of 32± 2°C/20± 2°C day/night, irradiance of 1000–1200 μmol m^−2^ s^-1^at full sun and a relative air humidity of 70%.

### Experimental design

The experiment consisted of four levels of soil alkalinity (0/0, 0/60, 0/180 and 0/300 mmol L^-1^) and two levels of rhizome connections (rhizome-connected and rhizome-severed treatments). Soil alkalinity treatment was conducted through alkaline solution with the mixture of Na_2_CO_3_ and NaHCO_3_ (sodium ions in a 1:1 ratio). Original ramets always experienced zero-addition of alkaline solution and subsequent ramets were always growing in soils with the alkaline solution addition levels of 0, 60, 180 or 300 mmol L^-1^. The experiment is a factorial design, and the eight treatments were coded as: 0C, 60C, 180C and 300C (0, 60, 180 or 300 mmol L^-1^ in the rhizome-connected treatment) and 0S, 60S, 180S and 300S for the rhizome-severed treatment. Each treatment was replicated 6 times. Altogether, we used 48 round plastic pots (26 cm in diameter and 17 cm in height) filled with vermiculite. The pots were separated into two compartments by a plastic divider across the middle of each pot. The plastic divider was waterproofed at the edges using silicone caulking. Rhizomes were fitted through a slit in the divider that was then sealed with tape.

We selected 48 uniform original ramets with rhizomes bearing more than one new subsequent ramets two months after germination. Original ramets were planted in one compartment and their subsequent ramets were planted on the other side. At the beginning of the experiment, the mean subsequent ramets height was 29.8 ± 1.95 cm, with ramets consisting of 6–7 leaves and connected to the original ramets by a common rhizome (roughly 10 cm length). After one week, the rhizome-severing treatment was conducted by severing the rhizome between original and subsequent ramets. Another week later, we applied four levels of 0/0, 0/60, 0/180 and 0/300 mmol L^-1^ alkaline solution into the compartment with original ramets. To reduce the potential osmotic shock for the 0/60, 0/180 and 0/300 alkalinity treatments, alkaline solution levels were increased daily by 30-mmol L^-1^ increments until the final concentrations were reached. Each pot were fertilized biweekly with 600 mL single strength Hoagland solution [34].

### Measurements

We recorded the tiller number and height every month from May to August 2013. Net photosynthetic rates (*P*_N_) of leaves were determined during 8:30–10:30 a.m on fully expanded first blades, using a portable open-flow, gas-exchange system (LI-6400, LICOR Biosciences, Lincoln, NE USA). Leaf water potential was measured using a Dewpoint PotentiaMeter (WP4-T, Decagon, Pullman, WA USA) between 6:00–6:45 a.m on the day before harvest. Rhizome length of subsequent ramets was determined using a PHOTO Scanner (Epson Perfection v700, Epson, China) and analysis software (WinRHIZO, Regent, Canada). All plant materials were oven-dried at 65°C for 72 hours before weighing.

Cost-benefits were determined according to Yu *et al*. (2001) as (b-c)/c ×100%, where c is the total biomass of clonal organs (a pair of original and subsequent ramets) in the severed treatment and b is the total biomass of clonal organs in the connected treatment. (b-c)/c × 100%> 0 indicates that the clonal plants benefited from clonal integration while (b-c)/c × 100% < 0 indicated that the clonal plants paid cost from clonal integration.

### Data analysis

A two-way ANOVA was used to investigate the effects of rhizome connection and alkalinity treatments and their interactions on the biomass of original and subsequent ramets, photosynthetic rates and water potential of subsequent ramets, as well as root weight, rhizome length and bud number. Repeated-measures ANOVA was employed to test the effects of rhizome connection, alkalinity treatments and date on tiller number and height of the subsequent ramets. Rhizome connection and alkalinity treatments were treated as fixed effects in these ANOVAs. These ANOVAs were followed by a T test so that the differences between the connected and severed treatment could be detected, while Duncan’s multiple-range test was used to compare the significance of the effects for the four alkalinity treatments. We used one-way ANOVA to analyze the effects of alkalinity treatments on cost-benefits of clonal integration. All data were checked for normality before analysis and none deviated significantly from a normal distribution. Statistical analyses were performed using SAS version 9.1 (SAS Institute, Cary, NC USA, 2002).

## Results

### Tiller number and height of subsequent ramets

**Fig 1:**
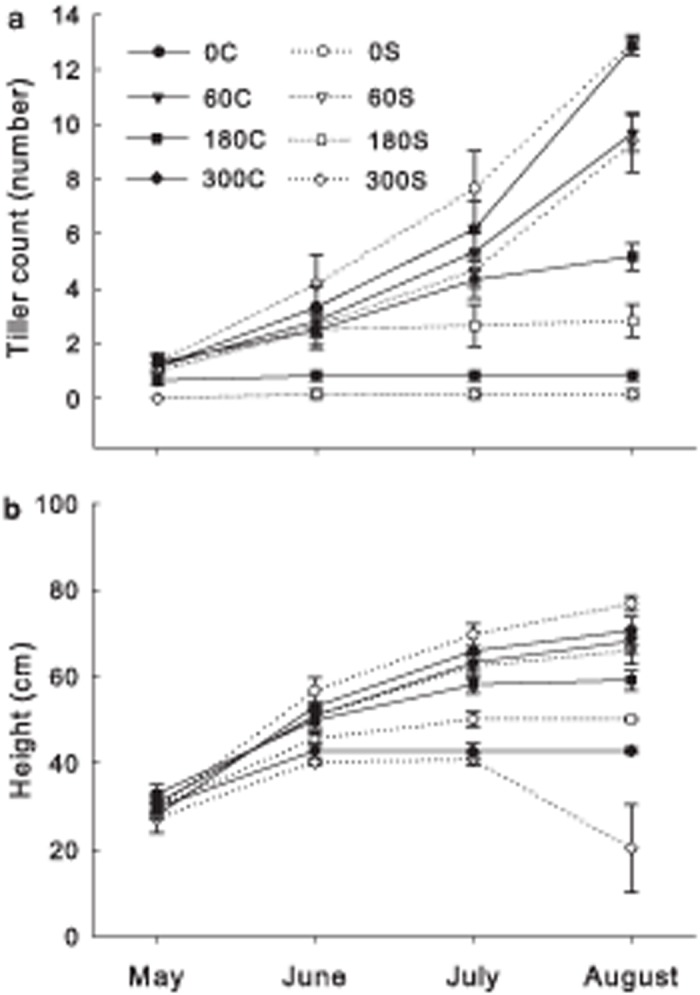
Effects of alkalinity stress, rhizome connection and data on the tiller number and height of subsequent ramets. Solid lines with 0C, 60C, 180C and 300C represented 0/0, 0/60, 0/180 and 0/300 mmol L^-1^ alkalinity levels in the rhizome-connected treatments, respectively; Dash lines with 0S, 60S, 180S and 300S represented 0/0, 0/60, 0/180 and 0/300 mmol L^-1^ alkalinity levels in the rhizome-severed treatments, respectively.

Both alkalinity treatment, date and their interactions had significant effects on the tiller number of subsequent ramets. The tiller number increased markedly as time extends under 0/0 and 0/60 alkalinity treatment in both rhizome-connection and rhizome-severed treatments and 0/180 in rhizome-connection treatment, but did not alter for the 0/180 in rhizome-connection treatment and 0/300 treatments (Table 1; Fig. 1a). The tiller number did not show significant response to rhizome-connection treatment (Table 1; Fig. 1a. At the end of the experiment, 50% of subsequent ramets in the rhizomes-severed treatment died under 0/300 alkalinity treatment, whereas all subsequent ramets in the corresponding rhizome-connected treatment survived (300C; Fig. 1a).

**Table 1:** Analysis of variance for the effects of alkalinity stress (A), rhizome connection (C) and data (D) on the tiller number and height of subsequent ramets.

	Source of variation							
		A	C	A×C	D	D×A	D×C	D×A×C
Variable	DF	3, 40	1, 40	3, 40	3, 93	9, 93	3, 93	9, 93
Tiller number		99.61***	2.05 ^ns^	2.28 ^ns^	180.69***	38.72***	0.81 ^ns^	0.76 ^ns^
Height		74.01***	6.47*	6.46**	263.8***	25.54 ***	3.34*	2.7**

Note: *F*-values are shown for each variable followed by their respective significance levels.

*P < 0.05;

**P < 0.01;

***P <0.001;

^ns^, *P* > 0.05.

Alkalinity treatment, severing, date and interaction of these three treatments had significant effects on the height of subsequent ramets (Table 1; Fig. 1b; *P* < 0.05). The height significantly decreased with increased alkalinity stress (Table 1; Fig. 1b). Rhizome connection did not affect the height of the subsequent ramets under the 0/0, 0/60 and 0/180 treatments (Fig. 1b; *P* > 0.05). However, the connected ramets were much taller than the severed ramets under the 0/300 alkalinity treatments (*P* < 0.05).

### Root weight, rhizome length, bud number and physiological features of subsequent ramets

**Fig 2:**
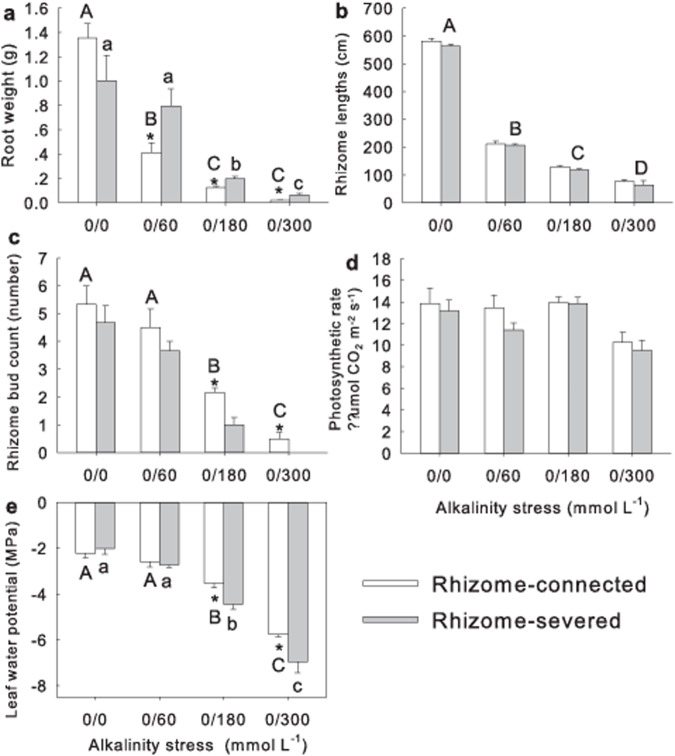
Effects of alkalinity stress and rhizome connection on a) root weight, b) rhizome length, c) bud number, d) photosynthetic rate and e) water potential of subsequent ramets. Bar groups with different capital letters indicate significant differences (*P* < 0.05) between the four levels of alkalinity in the rhizome-connected treatment. Bar groups with different lowercase letters indicate significant differences (*P* < 0.05) between the four levels of alkalinity in the rhizome-severed treatment. Asterisks indicate significant differences (*P* < 0.05) between rhizome-connected and rhizome-severed treatments.

The root weight of subsequent ramets significantly decreased with increases in alkalinity stress (Fig. 2a; *P* < 0.05). The connected ramets had considerably lower root weights than those for severed ramets with 0/60, 0/180 and 0/300 alkalinity treatments (Fig. 2a; *P* < 0.05). Rhizome length of the subsequent ramets decreased markedly as alkalinity stress rose (Table 2; Fig. 2b; *P* < 0.05). No differences were observed in rhizome length for the rhizome connection treatments (Fig. 2b; *P* > 0.05).

**Table 2:** Analysis of variance for the effects of alkalinity stress (A) and rhizome connection (C) on the photosynthetic rate, water potential, root weight, rhizome length and rhizome buds of subsequent ramets.

	Source of variation			
		A	C	A×C
Variable	DF	3, 40	1, 40	3, 40
Photosynthetic rate		7.39**	1.82^ns^	0.41^ns^
Water potential		114.1***	8.35**	3.46*
Root weight		49.9***	0.24^ns^	4.24*
Rhizome length		1288.97***	3.53^ns^	0.2^ns^
Rhizome buds		50.44***	6.56*	0.21^ns^

Note: *F*-values are shown for each variable followed by their respective significance levels.

*P < 0.05;

**P < 0.01;

***P <0.001;

^ns^, *P* > 0.05.

Alkalinity treatments markedly influenced the bud number of subsequent ramets with considerably lower number of buds seen as alkalinity stress rose (Table 2; Fig. 2c; *P* < 0.05). Rhizome connection treatment significantly increased the bud number at highly alkalinity levels (Table 2; Fig. 2c; *P* < 0.05). No rhizome buds survived in the 300S treatment, whereas an average of 0.5 buds survived in the corresponding 300C treatment (Fig. 2c).

Photosynthetic rates decreased markedly as alkalinity stress rose (Table 2; Fig. 2d; *P* < 0.05) with rhizome connectivity essentially having no effect (*P* > 0.05). Rhizome connection, alkalinity treatments and their interaction significantly affected the leaf water potential of the subsequent ramets (Table 2; Fig. 2e; *P* < 0.05). The leaf water potential decreased markedly as alkalinity stress rose (Fig. 2e; *P* < 0.05). The leaf water potential of subsequent ramets was unchanged between the connected and severed ramets for the 0/0 and 0/60 treatments (Fig. 2e; *P* > 0.05). However, the leaf water potential was markedly higher in the connected treatment compared to the severed treatment at higher alkalinity levels of 0/180 and 0/300 (Fig. 2e; *P* < 0.05).

### Biomass of daughter and original ramets

**Fig 3:**
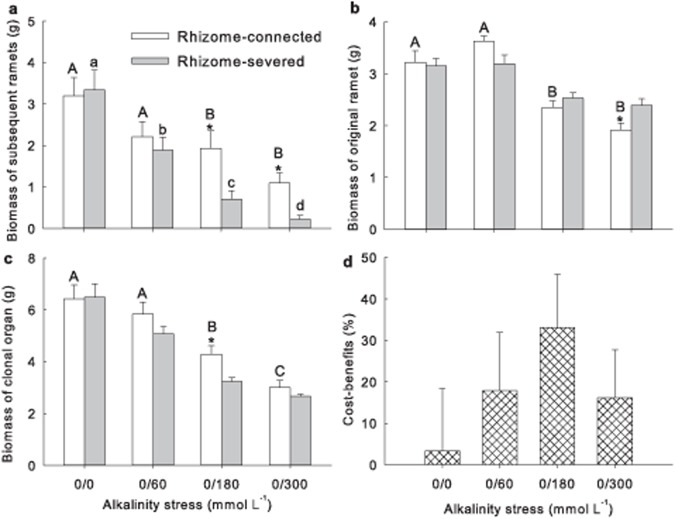
Effects of alkalinity stress and rhizome connection on a) the biomass of daughter and b) mother ramets, c) the total biomass of clonal organs and d) cost-benefits of clonal integration. Bar groups with different capital letters indicate significant differences (*P* < 0.05) between the four levels of alkalinity in the rhizome-connected treatment. Bar groups with different lowercase letters indicate significant differences (*P* < 0.05) between the four levels of alkalinity in the rhizome-severed treatment. Asterisks indicate significant differences (*P* < 0.05) between rhizome-connected and rhizome-severed treatments.

There was a significant interaction between rhizome connection and alkalinity treatments found for the biomass of subsequent ramets (Table 3; Fig. 3a; *P* < 0.05). Alkalinity treatment decreased markedly as alkalinity stress increased (*P* < 0.05). Biomass did not differ between the connected and severed ramets for the 0/0 and 0/60 treatments (*P* > 0.05), but was considerably greater for the connected ramets at higher alkalinity levels of 0/180 and 0/300 (*P* < 0.05).

**Table 3:** Analysis of variance for the effects of alkalinity stress (A) and rhizome connection (C) on the biomass of original and subsequent ramets and cost-benefit analysis.

	Source of variation				
			A	C	A×C
Variable		DF	3, 40	1, 40	3, 40
Subsequent ramet biomass			18.97***	14.13***	5.57**
Original ramet biomass			33.25***	0.16^ns^	3.42*
Cost and benefit			39.86***	3.97 ^ns^	0.88 ^ns^

Note: *F*-values are shown for each variable followed by their respective significance levels.

*P < 0.05;

**P < 0.01;

***P <0.001;

^ns^, *P* > 0.05.

The biomass of original ramets decreased considerably as the alkalinity stress rose (Fig. 3b; *P* < 0.05). There was no significant difference in the biomass between the connected and severed ramets in the 0/0, 0/60 and 0/180 treatments (Fig. 3b, *P* > 0.05). However, the biomass of connected original ramets decreased compared to severed rhizomes in the 0/300 treatment (Fig. 3b; *P* < 0.05).

### Cost and benefit

Cost-benefit analysis revealed considerable benefits of clonal integration for the clonal organs. The biomass of clonal organs decreased markedly with increases in alkalinity stress (Table 3; Fig. 3c; *P* < 0.05). No differences were observed in clonal organs between the rhizome connected and severed treatments under 0/0, 0/60 and 0/300 (Fig. 3c; *P* > 0.05), but the connected ramets had considerably greater biomass than those for severed ramets with 0/180 alkalinity treatment (*P* < 0.05). The benefits of clonal organs did not show significant response to alkalinity treatment (Fig. 3d; *P* > 0.05). The cost-benefits were greater than zero across the alkalinity treatments.

## Discussion

The experimental results showed that clonal integration markedly increased the performance of clonal plants under soil alkalinity stress. In the absence of clonal integration, the subsequent ramets died and the survival rate has fallen by half at the highest alkalinity level. Clonal integration was also found to ameliorate the negative effects of highly salinity stress [3, 7], drought [8] and nutrition deficiency [2, 9]. In our experiments, the biomass, leaf water potential, heightand buds of subsequent ramets in the rhizome-connected treatment was higher than that seen for the rhizome-severed treatment under highly alkalinity conditions (0/180 and 0/300 treatment). Besides, photosynthetic rates of the subsequent ramets did not show significant response to rhizome connection treatment. Thus, the higher biomass of subsequent ramets in the rhizome-connected treatment might be attributed to clonal integration, and subsequent ramets exposed to high alkaline conditions presumably received water and carbohydrates from their connected original ramets through clonal integration, which greatly mitigated the negative effects of alkalinity stress. Previous studies found that original ramets exposed to lower salinity could transporte water and carbon products to subsequent ramets growing in higher salinity, which reduced salinity stress in the connected subsequent ramets [10]. Our results suggest that clonal integration greatly contributes to the defenses of clonal plants against alkalinity stress.

Our study addressed the effects of clonal integration on clonal organs. The roots, rhizomes and buds of subsequent ramets showed different responses to clonal integration. Under the high alkaline conditions, the rhizome-connected treatment markedly increased the bud number, but showed negative effects on roots. These results indicated that the nutrients demand for the buds of subsequent ramets are presumably mainly supplied from the original ramets through clonal integration other than roots in highly alkalinity stress. When the rhizome was severed, subsequent ramets were required to allocate resources to root growth in order to acquire soil resources. These findings were consistent with a previous study, which showed that biomass allocation to roots decreased when the rhizomes were connected [35]. The rhizome length of subsequent ramets in our study appeared to increase but not significantly, which may be attributed to the restricted rhizome spread permitted by the pot dimensions. However, clonal integration had a significant positive effect on the buds of subsequent ramets, suggesting that clonal integration confers order on resource transfer, with resources from original ramets perhaps transporting with priority to buds of subsequent ramets positioned next to the roots.

Since clonal plants are propagated via below-ground rhizomes and buds, most original ramets resources may be transported to the rhizomes and buds of subsequent ramets, which could provide defenses against high-stress conditions and facilitate escape from sites where stress levels are intense [17, 35]. Other studies also showed that plants producing long linear rhizomes with little branching improved chances for progeny survival in highly stressful habitats [36–37]. This mechanism could be related to the fact that an integrated organization is a means by which resources can be conserved and recycled within clonal organs and may also have the advantage of concentrating resources in the most promising organs [35].

Clonal integration is influenced by environmental condition [20]. In this study, the clonal integration was detected under highly alkalinity levels (i.e. 0/180 and 0/300 treatments), but clonal integration presumably appears not to occur under low alkalinity level (0/60 treatment) and control group (0/0 treatment). This finding agrees with the notion that clonal integration is more important in instances of local stress conditions [38]. Original and subsequent ramets can experience contrasting alkalinity stress when the soil has differences in alkalinity, with subsequent ramets experiencing high alkaline conditions requiring higher levels of resource support from original ramets in order to survive in the more stressful conditions. These could result in an enhancement of integration processes within the whole clone through feedback regulation mechanisms. However, clonal integration appears not to occur under low alkalinity stress conditions (0/60 treatment), likely because the defense mechanisms of subsequent ramets could ensure its growth and clonal integration through rhizome was not necessary. Under homogeneous conditions (0/0 treatment), rhizome connection slightly decreased the subsequent ramets biomass, suggesting that rhizome connections might have a negative effect in homogeneous environments. These results were in agreement with previous studies that clonal integration was disadvantageous in homogeneous environments and advantageous in highly heterogeneous environments [23, 39]. In a homogeneous habitat, ramets occupied microhabitats with similar levels of external resource availability and net resource transfer between ramets would make their performance less equal, which made clonal integration disadvantageous in such habitats [39]. The ability to select clonal integration and ramets interconnectivity in heterogeneous alkaline environments were probably related to the functional plasticity that allowed performances to be adjusted in response to alkalinity[1].

Clonal integration can improve plants survival and reproduction but also pay a cost [26–27]. In this study, the benefits derived by subsequent ramets in high alkaline environments came at the expense of reduced growth in the supporting original ramets exposed to a non-alkaline environment, although the benefits received by subsequent ramets outweighed the costs incurred by their connected original ramets. Therefore, clonal integration confers a benefit to the whole clonal organs. This finding is somewhat consistent with other studies, which reported that subsequent ramets experiencing increased benefits without any cost to original ramets [4, 16] and clonal integration led to the transportation costs for original ramets [26–27]. Here, the net benefits received by the clonal organs through clonal integration increased by 3.4%, 17.9%, 33.1% and 13.1% in 0/0, 0/60, 0/180 and 0/300 treatment, respectively. In stressful environments, clonal plants allocated more resources to those organs that were currently affected by the stress-induced damage [17]. Clonal plants can optimize the efficiency of their resource utilization and defense systems by transporting resources between more favorably-placed ramets and ramets located in less-favorable microhabitats. Such integration might reasonably govern plant growth regulation and the phylogenetic microevolution of clonal plants exposed to stressful environments.

In conclusion, the present study suggests that clonal integration increases the adaptation of clonal plants to soils with alkalinity stress and affects the resource allocation pattern of clonal organs. Clonal integration may be selected for the habitats with highly alkalinity and do not occur in homogeneous habitats or those with low alkalinity. Cost-benefit analysis based on biomass measures shows that the original ramets incur a cost to enhance the survival of subsequent ramets but clonal integration confers overall benefits to the entire clonal organs. Therefore, clonal integration is an important adaptive strategy by which rhizomatous clonal plants can survive in alkalinity stress.
